# Device-measured movement behaviors and cardiac biomarkers in older adults without major cardiovascular disease: the Seniors-ENRICA-2 study

**DOI:** 10.1186/s11556-023-00313-8

**Published:** 2023-03-09

**Authors:** Blanca Fabre-Estremera, Antonio Buño-Soto, Esther García-Esquinas, Verónica Cabanas-Sánchez, David Martínez-Gómez, Fernando Rodríguez-Artalejo, Rosario Ortolá

**Affiliations:** 1 Department of Preventive Medicine and Public Health, Universidad Autónoma de Madrid, Calle del Arzobispo Morcillo 4, 28029 Madrid, Spain; 2 Department of Laboratory Medicine, La Paz University Hospital-IdiPaz, Paseo de la Castellana 261, 28046 Madrid, Spain; 3 National Centre for Epidemiology, Instituto de Salud Carlos III, Avenida Monforte de Lemos 3-5, 28029, Madrid, Spain; 4 CIBER of Epidemiology and Public Health (CIBERESP), Avenida Monforte de Lemos 3-5, 28029, Madrid, Spain; 5 IMDEA Food Institute. CEI UAM+CSIC, Carretera de Canto Blanco 8, 28049, Madrid, Spain

**Keywords:** High-sensitivity cardiac troponin T, NT-proBNP, Physical activity, Sedentary behavior, Older adults

## Abstract

**Background:**

High-sensitivity cardiac troponin T (hs-cTnT) and N-terminal pro-brain natriuretic peptide (NT-proBNP) are biomarkers of myocardial infarction and heart failure, respectively, and indicate cardiovascular risk. Since low physical activity (PA) and sedentary behavior (SB) are also associated with higher cardiovascular risk, and this association could be a consequence of higher levels of cardiac biomarkers, we examined the association of device-measured movement behaviors with hs-cTnT and NT-proBNP in older men and women without major cardiovascular disease (CVD).

**Methods:**

We used data from 1939 older adults from the Seniors-ENRICA-2 study. Accelerometers were used to assess time spent in sleep, SB, light PA (LPA), and moderate-to-vigorous PA (MVPA). Linear regression models were fitted separately in eight strata defined by sex, by median total PA time, and by the presence of subclinical cardiac damage according to cardiac biomarkers levels.

**Results:**

In the less active men with subclinical cardiac damage, spending 30 min/day more of MVPA was associated with a mean percentage difference (MPD) (95% confidence interval) in hs-cTnT of − 13.1 (− 18.3, − 7.5); MPDs in NT-proBNP per 30 min/day increment were 5.8 (2.7, 8.9) for SB, − 19.3 (− 25.4, − 12.7) for LPA and − 23.1 (− 30.7, − 14.6) for MVPA. In women with subclinical cardiac damage who were less physically active, 30 min/day more of SB, LPA and MVPA were associated with MPDs in hs-cTnT of 2.1 (0.7, 3.6), − 5.1 (− 8.3, − 1.7) and − 17.5 (− 22.9, − 11.7), respectively, whereas in those more active, LPA and MVPA were associated with MPDs of 4.1 (1.2, 7.2) and − 5.4 (− 8.7, − 2.0), respectively. No associations were found with NT-proBNP in women.

**Conclusions:**

The relationship between movement behaviors and cardiac biomarkers in older adults without major CVD depends on sex, subclinical cardiac damage and PA level. More PA and less SB were generally related to lower cardiac biomarkers levels among less active individuals with subclinical cardiac damage, with greater benefits for hs-cTnT in women than men and no benefits for NT-proBNP in women.

**Supplementary Information:**

The online version contains supplementary material available at 10.1186/s11556-023-00313-8.

## Introduction

Cardiovascular diseases (CVD) are the leading cause of death worldwide, especially among individuals > 65 years, where they accounted for 42% of all deaths in 2019 [1]. While serum cardiac troponins and natriuretic peptides are useful for the clinical management of acute myocardial infarction and heart failure, respectively, in the general population, these two biomarkers are related to subclinical cardiac damage and indicate CVD risk [2–4].

Cardiac troponin (cTn) is a heterotrimeric complex (troponin T, troponin C and troponin I) that regulates the interaction between actin and myosin filaments in cardiac muscle. As cTnT and cTnI are highly specific for cardiac myocytes, both have become the standard biomarkers for risk stratification in patients with suspected acute coronary syndrome and for the diagnosis of myocardial infarction [5, 6]. An important step forward has been the development of high-sensitivity assays, which detect concentrations 10–100 times lower than those of conventional assays. The clinical decision value for acute myocardial infarction is the 99th percentile (p99) of the reference population, preferably stratified by sex, which facilitates earlier treatment or exclusion resulting in better outcomes [5–7].

The most commonly used biomarkers for the diagnosis of heart failure and cardiac dysfunction are the B-type natriuretic peptides, mostly synthesized and secreted by left ventricle myocytes: N-terminal pro-B-type natriuretic peptide (NT-proBNP) and biologically active B-type natriuretic peptide. The recommended cut-off values to exclude heart failure in a non-acute setting are 35 and 125 pg/mL for BNP and NT-proBNP, respectively. In addition, an age-dependent cutoff value of NT-proBNP may be more useful in this setting [8, 9].

Physical inactivity has been consistently associated with a significant increase in CVD risk and a decrease in life expectancy [10–12]. In older adults, total physical activity (PA) has been shown to have a favorable impact on metabolic disease [13], hypertension [14], premature mortality [15] and leukocyte telomere length [16], a hallmark of aging. Moderate-to-vigorous PA (MVPA) has also been associated with significant protection from coronary heart disease (CHD) [17, 18]. Conversely, sedentary behavior (SB) has been inversely associated with all-cause mortality, CVD mortality and cancer mortality, and also with a lower incidence of CVD, cancer and type-2 diabetes in older adults, independently of PA [19]. Additionally, sleep duration has been related to CVD risk [20, 21]. Therefore, a balance between different movement behaviors (sleep, SB and different intensities of PA) is strongly encouraged [22].

Most previous research on the relationship between movement behaviors and cardiac biomarkers has only focused on PA in younger active individuals, reporting exercise-induced troponin elevations above the p99 [23, 24]. However, few studies have investigated the association between movement behaviors and cardiac biomarkers in older adults [25–27]. This is important because such association could explain the effect of movement behaviors on CVD risk in old age. To our knowledge, only one study has analyzed cardiac biomarkers concentrations in older adults with different levels of movement behaviors, finding that MVPA may be more important in protecting against cardiac health deterioration in less active individuals [27]. However, the mentioned study only included men and did not consider 24-hour movement behaviors, including sleep, or other accelerometry variables of interest, such as bouted time or mean movement intensity. Also, as with many preventive interventions, the effect of PA may depend on the level of cardiac damage or CVD risk [28]. Therefore, we aimed to investigate the association between device-measured movement behaviors and serum high-sensitivity cardiac troponin T (hs-cTnT) and NT-proBNP in older men and women without major CVD. We hypothesized that this association depends on baseline levels of cardiac biomarkers as well as the level of PA.

## Materials and methods

### Study design and participants

Data came from the Seniors-ENRICA-2 cohort [29]. Participants were recruited between 2015 and 2017 by stratified random sampling of all community-dwelling individuals aged 65 years and older holding a national healthcare card and living in two districts of the city of Madrid (Spain) and four large surrounding towns. Initially, a computer-assisted telephone interview was conducted to collect socio-demographic, lifestyle and morbidity data. Next, two home visits by study staff were done to perform a physical examination, obtain a diet history, place a wrist accelerometer, and obtain serum samples.

### Study variables

#### Device-measured movement behaviors

Each participant in the study received an ActiGraph GT9X accelerometer (ActiGraph Inc., Pensacola, FL, USA), and was asked to wear it on their non-dominant wrist (to minimize misclassification of arm movements during sedentary activities as physical activity) for seven consecutive days without removing it unless it was for bathing or swimming. Details on the processing of the accelerometer data have been published elsewhere [30]. The raw accelerometer data were processed using the GGIR package (v.1.7–0, https://cran.r-project.orgweb/packages/GGIR/) in R [31].

SB and PA intensities were identified using previously proposed thresholds for the Euclidean Norm of the raw accelerations Minus One (ENMO), in milligravitational units (mg): < 45 mg for SB, 45–99 mg for light PA (LPA), and ≥ 100 mg for MVPA [32]. Sleep periods were detected with an automatized algorithm [33]. Total PA time was the result of the sum of time in LPA and MVPA. Time in sedentary bouts ≥30 min, and in MVPA bouts ≥10 min were also registered, considering bouts in each behavior when 80% of the minimum required time met the threshold criteria. The number of sedentary breaks was estimated by subtracting 1 to the number of sedentary blocks, regardless of duration. Mean movement intensity was estimated with the daily mean of acceleration in mg. To avoid SB and PA underestimation [34], participants were included if they had at least 4 valid days (≥3 weekdays and ≥ 1 weekend-day), in which they wore the accelerometer ≥16 h/day. Non-wear time and time with abnormally high accelerations (i.e., ≥5.5 g) were imputed using the mean of the acceleration recorded for each participant during the corresponding time intervals.

#### Cardiac biomarkers

Fasting venous blood samples were collected from the arm of each participant in RST tubes with thrombin-based clot activator and polymer gel (Becton Dickinson). The tubes were centrifuged at 3.000 rpm for 10 minutes within 3 h of collection and serum was aliquoted, frozen at − 80 °C and stored up to 3.6 years at the Department of Preventive Medicine and Public Health, Universidad Autónoma de Madrid. Serum hs-cTnT and NT-proBNP were measured between July 2019 and June 2020 on a cobas®6000 analyzer (Roche Diagnostics) using an electrochemiluminescence Elecsys® immunoassay, at the Department of Laboratory Medicine, ‘La Paz’ University Hospital (Madrid). The hs-cTnT and the NT-proBNP assays have a limit of detection of 3 pg/mL and 10 pg/mL, respectively. The assays were performed using the manufacturer’s calibrators and quality controls. For hs-cTnT, the inter-assay coefficient of variation was 5.10% for a mean concentration of 26.86 pg/mL and 3.60% for a mean concentration of 2000.96 pg/mL. For NT-proBNP, the inter-assay coefficient of variation was 7.28% for a mean concentration of 134.92 pg/mL and 8.33% for a mean concentration of 4609.34 pg/mL. The Roche hs-cTnT assay has a sex-specific 99th percentile upper reference limit (URL) of 9.0 ng/L for females and 16.8 ng/L for males.

#### Potential confounders

We also collected information on sociodemographic and lifestyle characteristics, including sex, age, educational level, tobacco smoking and alcohol consumption. Food consumption and energy intake (kcal/day) were obtained from a validated diet history [35]; the diet quality was estimated with the Mediterranean Diet Adherence Screener (MEDAS), which ranges from 0 to 14, with higher scores indicating higher quality [36]. Height and weight were measured by trained staff under standardized conditions using electronic scales (model Seca 841, precision to 0.1 kg) and portable extendable stadiometers (model Ka We 44444Seca), with the participants barefoot and lightly clothed, and body mass index (BMI) was calculated as weight (kg) divided by height (m) squared. Systolic blood pressure (SBP) in mmHg was measured three times separated by 1–2-minute intervals, by trained study staff under standardized conditions with validated automatic devices (Omron model M6), using the mean of the second and third measurements for analyses. Fasting serum glucose, total cholesterol, triglycerides and creatinine were measured on Atellica® Solution-CH chemistry analyzer (Siemens Healthineers) using colorimetric enzymatic methods. LDL-cholesterol measurement depended on triglycerides levels: if triglycerides < 250 mg/dL, LDL-cholesterol was calculated with the Friedewald formula (LDL = total cholesterol - triglycerides/5 - HDL), and if triglycerides ≥250 mg/dL, LDL-cholesterol was determined on Atellica® Solution-CH chemistry analyzer (Siemens Healthineers) by a colorimetric enzymatic method [37]. The estimated glomerular filtration rate (eGFR) was calculated with the Chronic Kidney Disease - Epidemiology Collaboration (CKD-EPI) eq. [38], and CKD defined as an eGFR < 60 mL/min/1.73m^2^. Lastly, the presence of major CVD was determined by medical diagnosis of acute myocardial infarction, stroke, chronic heart failure or atrial fibrillation recorded in the Primary Care database from the Community of Madrid (Spain).

### Statistical analysis

Analyses were performed separately in men and women, and by PA and subclinical cardiac damage, as these two variables also modified the study associations. Participants were classified as less or more active according to median total PA time (3.53 h/day) and the presence or absence of subclinical cardiac damage, determined by high baseline levels of hs-cTnT and/or NT-proBNP. The cutoff values used for hs-cTnT were based on the Fourth Universal Definition of Myocardial Infarction, which considers that the term myocardial injury should be used when there is evidence of elevated cTn values with at least one value above the sex-specific p99 URL (for the Roche hs-cTnT assay, in men: 16.8 pg/dL, in women: 9.0 pg/mL) [7]; those for NT-proBNP were based on the European Society of Cardiology guidelines, which consider levels ≤75 pg/mL if aged 65–75 years, and ≤ 250 pg/mL if > 75 years, to exclude heart failure in non-acute settings [8, 9].

Cardiac biomarker levels according to study participant characteristics were summarized with geometric means and geometric standard deviation factors, as their distributions were positively skewed. Associations between time in each movement behavior and hs-cTnT or NT-proBNP were analyzed by linear regression with log-transformed cardiac biomarker levels to achieve parametric distributions. Results were summarized with mean percentage differences (MPD), and their 95% confidence interval (CI), in cardiac biomarkers per 30 min/day increments in sleep, SB, LPA or MVPA, which were obtained by subtracting 1 from the exponentiated β coefficients in the regression models, and multiplying the result by 100. Three models were built with incremental adjustment for potential confounders: Model 1 adjusted for sex, age, and educational level; Model 2 further adjusted for tobacco smoking, alcohol consumption, MEDAS score, and energy intake; and Model 3 further adjusted for BMI, SBP, serum glucose and LDL-cholesterol levels, and eGFR. Since the results from the three models were very similar, only the fully adjusted ones are presented. Also, dose-response associations were evaluated by modeling time in each movement behavior as restricted cubic splines, with models for sleep and SB adjusted for MVPA time, and models for LPA and MVPA adjusted for SB time. *P* values for non-linearity were calculated by testing the null hypothesis that the coefficient of the second spline equals 0 using Wald tests. Associations of the other accelerometry variables with hs-cTnT or NT-proBNP levels were examined by modeling 30 min/day increments (for time in bouts) or 1-SD (standard deviation) increments (for number of sedentary breaks and mean movement intensity), using the same statistical procedures.

To account for potential false-positive results due to multiple testing, we calculated the 5% false discovery rate for all the comparisons using the Benjamini-Hochberg procedure [39], and adjusted the statistical significance accordingly.

Analyses were performed with Stata®, version 16 (StataCorp. 2019. Stata Statistical Software: Release 16. College Station, TX:StataCorp LLC).

## Results

From the 3.273 study participants in the Seniors-ENRICA-2 study, we excluded 643 with a previous diagnosis of major CVD and 8 without information about it, 586 without valid accelerometry records (478 without accelerometry measurements and 108 not meeting the wearing time requirements), 46 without hs-cTnT and/or NT-proBNP determinations, and 51 who lacked data on potential confounders. Thus, the analytical sample included 1.939 individuals.

Study participants had a mean age of 71.5 years and 55.65% were women. Supplemental Table 1 and Table 1 show the characteristics of study participants and cardiac biomarker concentrations in each stratum, respectively. Cardiac marker levels were higher in the less active participants than in the more active ones (Table 1). hs-cTnT levels were higher in men, older participants, and those with higher energy intake, higher SBP, higher glycaemia, lower LDL-cholesterol, and CKD, whereas NT-proBNP levels were higher in older participants and those with normal weight, higher glycaemia and CKD.

**Table 1:** Cardiac biomarkers by characteristics of study participants, stratified by subclinical cardiac damage and PA time

	hs-cTnT (pg/mL)								NT-proBNP (pg/mL)							
	No subclinical cardiac damage^**a**^				Subclinical cardiac damage^**a**^				No subclinical cardiac damage^**a**^				Subclinical cardiac damage^**a**^			
	Low PA time^**b**^		High PA time^**b**^		Low PA time^**b**^		High PA time^**b**^		Low PA time^**b**^		High PA time^**b**^		Low PA time^**b**^		High PA time^**b**^	
	n	GM (GSD)	n	GM (GSD)	n	GM (GSD)	n	GM (GSD)	n	GM (GSD)	n	GM (GSD)	n	GM (GSD)	n	GM (GSD)
Total	438	8.19 (1.36)	452	7.76 (1.36)	532	12.22 (1.63)	517	11.23 (1.55)	438	50.43 (1.70)	452	45.96 (1.64)	532	127.57 (1.99)	517	120.90 (1.78)
Sex																
Men	267	9.33 (1.31)	234	9.21 (1.32)	190	13.61 (1.66)	169	12.33 (1.54)	267	47.55 (1.68)	234	42.85 (1.65)	190	132.19 (2.17)	169	121.52 (1.88)
Women	171	6.69 (1.23)	218	6.54 (1.25)	342	10.09 (1.56)	348	8.83 (1.50)	171	55.29 (1.72)	218	49.56 (1.62)	342	125.07 (1.88)	348	120.61 (1.73)
Age (years)																
65–70	189	7.89 (1.33)	284	7.44 (1.36)	174	9.74 (1.54)	233	8.57 (1.49)	189	41.94 (1.46)	284	42.01 (1.57)	174	111.51 (1.85)	233	115.32 (1.66)
≥70	249	8.43 (1.34)	168	8.46 (1.33)	358	12.03 (1.65)	284	11.02 (1.56)	249	58.01 (1.80)	168	53.51 (1.70)	358	136.19 (2.03)	284	125.69 (1.87)
Educational level																
≤ Primary	248	8.18 (1.34)	289	7.83 (1.35)	343	11.53 (1.69)	352	9.89 (1.55)	248	51.69 (1.74)	289	47.12 (1.62)	343	126.21 (2.01)	352	122.04 (1.82)
Secondary	97	8.30 (1.35)	83	7.69 (1.40)	90	11.10 (1.53)	89	9.82 (1.57)	97	48.62 (1.60)	83	42.13 (1.71)	90	127.95 (2.15)	89	116.88 (1.69)
University	93	8.12 (1.32)	80	7.84 (1.36)	99	10.34 (1.52)	76	9.66 (1.58)	93	49.06 (1.71)	80	45.99 (1.65)	99	132.06 (1.74)	76	120.47 (1.74)
Tobacco smoking																
Non-smoker	202	7.80 (1.35)	215	7.44 (1.33)	316	10.93 (1.65)	310	9.68 (1.55)	202	54.79 (1.73)	215	45.59 (1.65)	316	126.11 (1.98)	310	119.85 (1.84)
Former smoker	180	8.61 (1.32)	200	8.11 (1.36)	165	11.79 (1.59)	168	9.95 (1.60)	180	46.33 (1.66)	200	45.75 (1.62)	165	122.20 (1.94)	168	115.74 (1.65)
Current smoker	56	8.35 (1.35)	37	8.35 (1.41)	51	11.29 (1.62)	39	10.75 (1.44)	56	49.14 (1.70)	37	49.45 (1.74)	51	157.46 (2.12)	39	156.44 (1.78)
Alcohol consumption																
Non- drinker	76	7.39 (1.29)	66	7.14 (1.28)	132	11.60 (1.75)	103	9.22 (1.53)	76	57.55 (1.75)	66	50.31 (1.53)	132	136.81 (2.03)	103	117.04 (1.66)
Former drinker	21	8.17 (1.36)	22	7.68 (1.25)	39	13.16 (1.99)	33	10.70 (1.52)	21	49.54 (1.74)	22	47.08 (1.59)	39	140.49 (2.10)	33	117.79 (1.66)
Moderate drinker^c^	238	8.29 (1.35)	240	7.78 (1.38)	268	10.76 (1.54)	264	9.94 (1.54)	238	49.71 (1.68)	240	46.70 (1.63)	268	123.14 (1.96)	264	117.10 (1.77)
Heavy drinker	103	8.60 (1.34)	124	8.26 (1.37)	93	11.33 (1.56)	117	9.96 (1.62)	103	47.46 (1.68)	124	42.31 (1.73)	93	122.83 (1.94)	117	134.69 (1.94)
MEDAS score (0–14)																
≤6	164	8.15 (1.35)	137	7.70 (1.36)	184	11.64 (1.75)	173	9.68 (1.57)	164	48.79 (1.75)	137	45.71 (1.63)	184	125.48 (2.00)	173	118.97 (1.76)
7–8	186	8.16 (1.33)	189	7.74 (1.35)	256	11.01 (1.57)	232	9.88 (1.56)	186	51.30 (1.71)	189	47.20 (1.57)	256	128.38 (1.97)	232	118.42 (1.75)
≥9	88	8.35 (1.34)	126	8.02 (1.37)	92	11.04 (1.56)	112	10.02 (1.54)	88	51.75 (1.59)	126	44.44 (1.77)	92	129.54 (2.02)	112	129.40 (1.87)
Energy intake (kcal/day)																
< 1800	120	7.29 (1.32)	130	6.92 (1.30)	227	10.77 (1.76)	180	8.86 (1.48)	120	56.93 (1.81)	130	45.75 (1.58)	227	129.81 (2.04)	180	121.83 (1.72)
180–2050	161	8.34 (1.34)	137	7.62 (1.36)	162	11.54 (1.56)	198	10.05 (1.55)	161	48.77 (1.69)	137	47.06 (1.65)	162	131.95 (1.97)	198	123.43 (1.85)
> 2050	157	8.79 (1.33)	185	8.64 (1.35)	143	11.62 (1.50)	139	10.94 (1.63)	157	47.58 (1.61)	185	45.32 (1.69)	143	119.44 (1.91)	139	116.24 (1.76)
BMI (kg/m^2^)																
< 25	103	8.07 (1.35)	138	7.58 (1.35)	127	10.92 (1.65)	172	9.40 (1.51)	103	56.11 (1.65)	138	48.47 (1.68)	127	133.58 (1.92)	172	127.01 (1.70)
25–30	227	8.28 (1.34)	237	7.85 (1.36)	232	10.99 (1.65)	232	10.30 (1.59)	227	50.39 (1.68)	237	45.80 (1.62)	232	121.96 (1.95)	232	115.65 (1.86)
≥30	108	8.14 (1.34)	77	8.08 (1.37)	173	11.78 (1.59)	173	9.59 (1.56)	108	45.63 (1.78)	77	42.25 (1.66)	173	131.00 (2.08)	173	123.31 (1.73)
SBP (mmHg)																
< 130	178	8.13 (1.37)	201	7.50 (1.37)	201	10.76 (1.63)	226	9.57 (1.52)	178	52.74 (1.73)	201	44.37 (1.66)	201	120.68 (1.88)	226	114.91 (1.74)
≥130	260	8.23 (1.32)	251	8.06 (1.34)	331	11.52 (1.63)	291	10.06 (1.59)	260	48.91 (1.68)	251	47.29 (1.63)	331	131.94 (2.04)	291	125.77 (1.81)
Serum glucose (mg/dL)																
< 100	267	7.96 (1.35)	302	7.64 (1.37)	339	10.71 (1.59)	380	9.53 (1.53)	267	52.76 (1.72)	302	47.04 (1.62)	339	132.41 (1.93)	380	122.67 (1.74)
≥100	171	8.58 (1.33)	150	8.15 (1.33)	193	12.20 (1.69)	137	10.76 (1.63)	171	47.00 (1.67)	150	43.87 (1.69)	193	119.49 (2.07)	137	116.14 (1.89)
Serum LDL-cholesterol (mmol/L)																
< 130	319	8.39 (1.35)	286	7.91 (1.37)	381	11.75 (1.63)	339	10.31 (1.58)	319	51.69 (1.72)	286	45.81 (1.69)	381	132.71 (2.00)	339	119.61 (1.79)
≥130	119	7.69 (1.32)	166	7.63 (1.33)	151	10.00 (1.62)	178	9.01 (1.50)	119	47.20 (1.66)	166	46.24 (1.57)	151	115.47 (1.93)	178	123.41 (1.77)
eGFR^d^ (mL/min/1.73m^2^)																
≥60	427	8.18 (1.34)	444	7.77 (1.36)	481	10.72 (1.58)	489	9.75 (1.56)	427	50.30 (1.70)	444	45.80 (1.64)	481	123.22 (1.94)	489	120.04 (1.77)
< 60	11	8.81 (1.37)	8	10.11 (1.18)	51	17.29 (1.81)	28	11.70 (1.49)	11	55.66 (1.63)	8	56.07 (1.75)	51	176.98 (2.21)	28	137.13 (1.90)

*BMI* body mass index, *eGFR* estimated Glomerular Filtration Rate, *GM* geometric mean, *GSD* geometric standard deviation factor, *hs-cTnT* high-sensitivity cardiac troponin T, *MEDAS* Mediterranean Diet Adherence Screener, *NT-proBNP* N-terminal pro-B-type natriuretic peptide, *PA* physical activity, *SBP* systolic blood pressure

^a^Subclinical cardiac damage: hs-cTnT >p99 (16.8 pg/mL in men and 9.0 pg/mL in women) and/or NT-proBNP >cutoff (75 pg/mL if age ≤ 75 years, 250 pg/mL if age > 75 years)

^b^Low PA time: total PA time ≤ 3.53 h/day; high PA time: total PA time > 3.53 h/day

^c^Moderate drinker: < 10 g/day in women and < 20 g/day in men

^d^Estimated Glomerular Filtration Rate by the CKD-EPI (Chronic Kidney Disease Epidemiology Collaboration) equation

Participants wore the accelerometer for a mean (SD) time of 22.83 (2.17) hours per day during 6.67 (0.64) valid days. Supplemental Table 2 shows time spent in each movement behavior among men and women in each stratum. Men spent more time in SB and MVPA and less time sleeping and in LPA than women. Less active participants (those with a total PA time ≤ 3.53 h/day) spent more time sleeping (men, 33%; women, 34%) and in SB (men, 57%; women, 56%), and less time in LPA (men, 7%; women, 9%) and MVPA (men, 3%; women, 2%) than the more active participants (31, 32, 50, 49, 12, 14, 7 and 6%, respectively). Compared with participants without subclinical cardiac damage, those with it spent more time in LPA (men only) and less time in MVPA (women only).

**Fig. 1:**
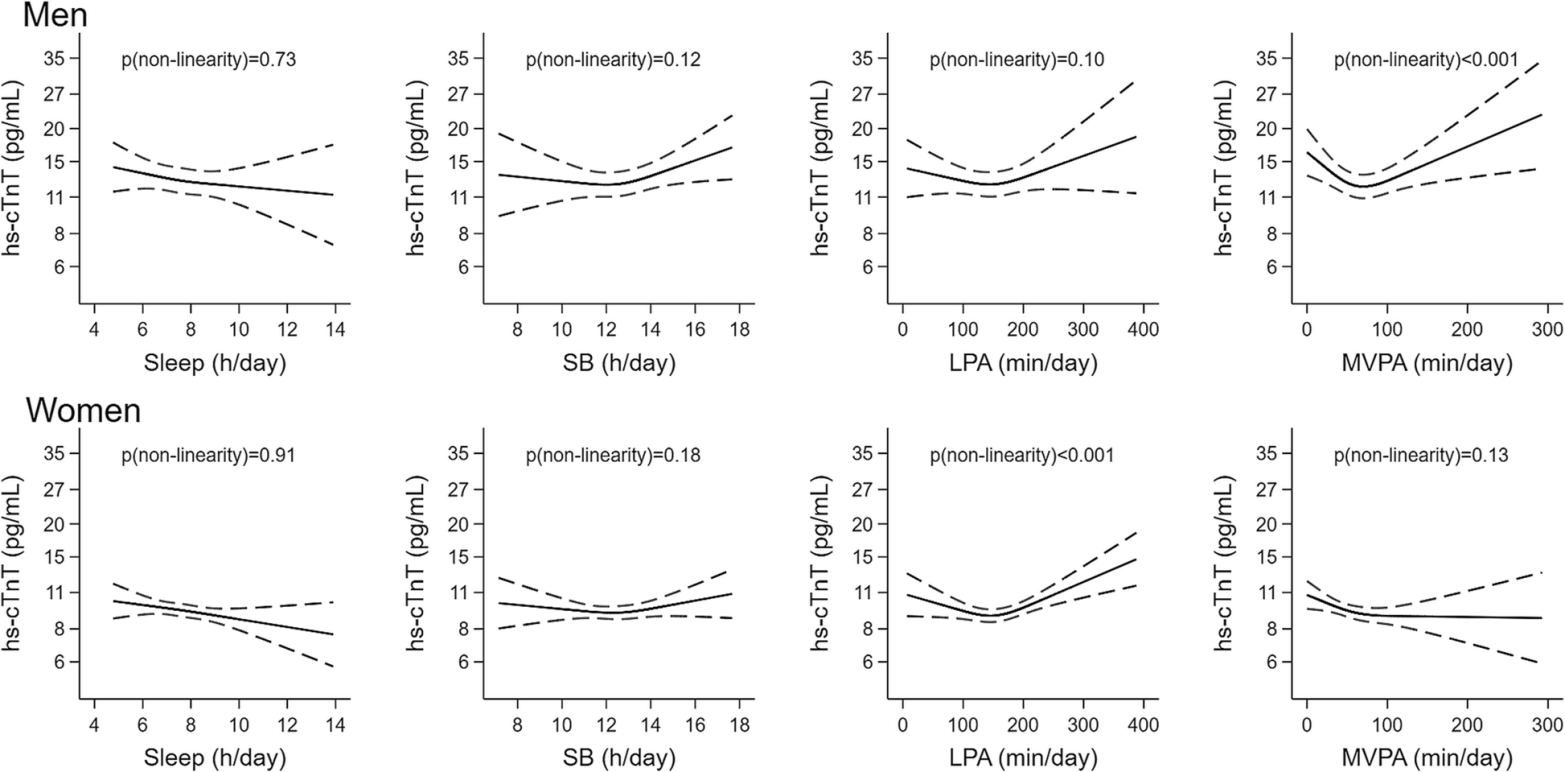
**Association of each movement behavior with hs-cTnT in men and women with subclinical cardiac damage.** Restricted cubic splines whose values are geometric means (95% confidence interval) of hs-cTnT hs-cTnT: high-sensitivity cardiac troponin T; LPA: light physical activity; MVPA: moderate-to-vigorous physical activity; PA: physical activity; SB: sedentary behavior Subclinical cardiac damage: hs-cTnT >p99 (16.8 pg/mL in men and 9.0 pg/mL in women) and/or NT-proBNP >cutoff (75 pg/mL if age ≤ 75 years, 250 pg/mL if age > 75 years). Linear regression models adjusted for sex, age, educational level (primary or less, secondary, or university), smoking status (never, former, or current), alcohol consumption (never, moderate, heavy, or former), energy intake (kcal/day), Mediterranean Diet Adherence Screener (MEDAS) score, body mass index (kg/m^2^), serum glucose (mg/dL), serum LDL-cholesterol (mg/dL), systolic blood pressure (mmHg) and glomerular filtration rate. Models for sleep and SB further adjusted for MVPA time, and models for LPA and MVPA further adjusted for SB time

**Fig. 2:**
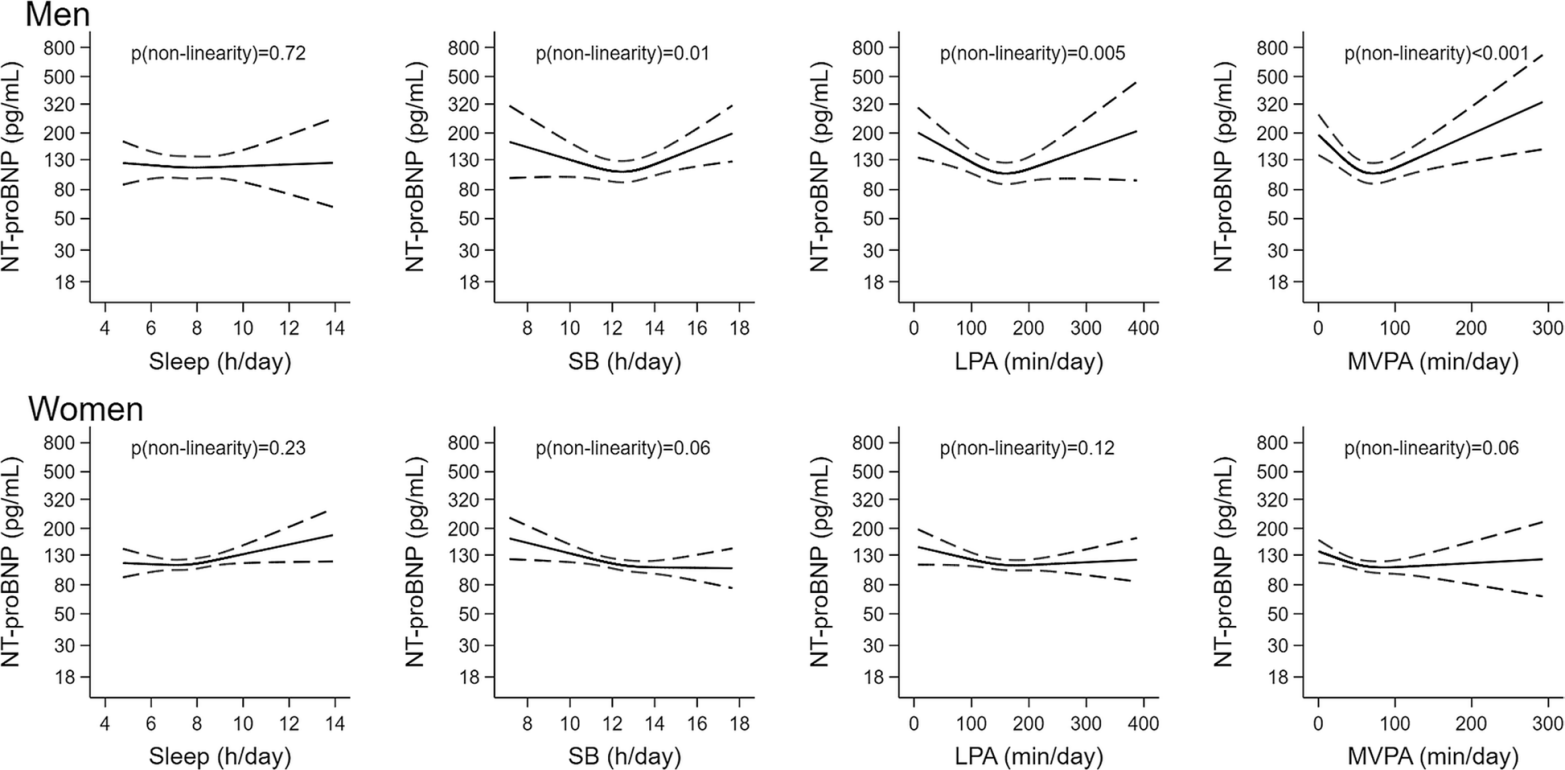
**Association of each movement behavior with NT-proBNP in men and women with subclinical cardiac damage.** Restricted cubic splines whose values are geometric means (95% confidence interval) of NT-proBNP LPA: light physical activity; MVPA: moderate-to-vigorous physical activity; NT-proBNP: N-terminal pro-B-type natriuretic peptide; PA: physical activity; SB: sedentary behavior Subclinical cardiac damage: hs-cTnT >p99 (16.8 pg/mL in men and 9.0 pg/mL in women) and/or NT-proBNP >cutoff (75 pg/mL if age ≤ 75 years, 250 pg/mL if age > 75 years). Linear regression models adjusted for sex, age, educational level (primary or less, secondary, or university), smoking status (never, former, or current), alcohol consumption (never, moderate, heavy, or former), energy intake (kcal/day), Mediterranean Diet Adherence Screener (MEDAS) score, body mass index (kg/m^2^), serum glucose (mg/dL), serum LDL-cholesterol (mg/dL), systolic blood pressure (mmHg) and glomerular filtration rate. Models for sleep and SB further adjusted for MVPA time, and models for LPA and MVPA further adjusted for SB time

Associations of time in each movement behavior with cardiac biomarkers in men and women with and without subclinical cardiac damage are shown with restricted cubic splines in Figs. 1 and 2 and Supplemental Figs. 1–2. Among participants with subclinical cardiac damage, there was evidence of departure from linearity in the associations with hs-cTnT of MVPA in men (*p* < 0.001) and of LPA in women (*p* < 0.001) (Fig. 1), and in the associations with NT-proBNP, of SB, LPA and MVPA in men (*p* = 0.010, *p* = 0.005 and *p* < 0.001, respectively) (Fig. 2). Such evidence was not found in participants without subclinical cardiac damage, except between LPA and NT-proBNP in men (*p* = 0.031) (Supplemental Figs. 1–2). The fact that study associations varied between males and females, between those with and without subclinical cardiac damage, and that it was not linear across PA level, further supported our decision to stratify the main analyses by sex, subclinical cardiac damage and PA level.

Table 2 and Supplemental Table 3 summarize the association of increments in each movement behavior with cardiac biomarkers in men and women, stratified by subclinical cardiac damage and PA time. Among men, no associations were found between movement behaviors and cardiac biomarker levels, except in the group of less active men with subclinical cardiac damage, where all movement behaviors except sleep were related with cardiac biomarkers levels. Thus, in this group, spending 30 min/day more in SB was related to higher NT-proBNP levels, with a MPD (95% CI) of 5.8 (2.7, 8.9) (Table 2), and accumulating SB time in bouts ≥30 min did not modify much the association (Supplemental Table 3). There was also an inverse association of the number of sedentary breaks with NT-proBNP (MPD per 1-SD increment of − 25.4 [− 33.1, − 16.8]), but not with hs-cTnT (Supplemental Table 3). Also in this group of less active men with subclinical cardiac damage, more LPA was linked to lower NT-proBNP levels (− 19.3 [− 25.4, − 12.7] per 30 min/day increment in LPA) and more time in MVPA was associated with lower levels of both cardiac biomarkers (− 13.1 [− 18.3, − 7.5] per 30 min/day increment for hs-cTnT and − 23.1 [− 30.7, − 14.6] for NT-proBNP) (Table 2). Accumulating MVPA time in bouts ≥10 min did not modify much the association with NT-proBNP levels (− 23.6 [− 37.2. -7.0]), but the association with hs-cTnT was greatly reduced and became non-significant (Supplemental Table 3). Lastly, a strong inverse association was found between mean movement intensity and NT-proBNP levels (− 27.8 [− 35.9, − 18.6] per 1-SD increment), but not with hs-cTnT (Supplemental Table 3).

**Table 2:** Association of movement behaviors with cardiac biomarkers in men and women, stratified by subclinical cardiac damage and PA time

	Men				Women			
	No subclinical cardiac damage^**b**^		Subclinical cardiac damage^**b**^		No subclinical cardiac damage^**b**^		Subclinical cardiac damage^**b**^	
	Low PA time^**c**^ *n* = 267	High PA time^**c**^ *n* = 234	Low PA time^**c**^ *n* = 190	High PA time^**c**^ *n* = 169	Low PA time^**c**^ *n* = 171	High PA time^**c**^ *n* = 218	Low PA time^**b**^ *n* = 342	High PA time^**c**^ *n* = 348
hs-cTnT								
Sleep	− 0.6 (− 2.4, 1.3)	− 0.4 (− 2.7, 1.9)	− 1.7 (− 3.6, 0.2)	− 0.9 (− 3.6, 1.9)	0.1 (− 2.2, 2.4)	− 0.4 (− 3.0, 2.2)	− 0.4 (− 2.0, 1.2)	− 2.3 (− 4.1, − 0.4)
SB	0.2 (− 1.4, 1.9)	0.1 (− 1.8, 2.0)	3.3 (1.4, 5.1)	− 0.7 (− 2.9, 1.4)	− 1.1 (− 3.4, 1.2)	− 1.0 (− 3.0, 0.9)	2.1 (0.7, 3.6)^d^	− 1.5 (− 0.1, 3.1)
LPA	1.9 (− 2.4, 6.5)	1.0 (− 3.1, 5.2)	− 3.9 (− 8.4, 0.8)	2.3 (− 1.9, 6.7)	2.2 (− 2.7, 7.3)	2.8 (− 0.8, 6.6)	− 5.1 (− 8.3, − 1.7)^d^	4.1 (1.2, 7.2)^d^
MVPA	− 1.1 (− 7.0, 5.3)	− 0.2 (− 4.0, 3.8)	− 13.1 (− 18.3, − 7.5)^d^	3.4 (− 1.3, 8.2)	6.4 (− 2.3, 16.0)	1.8 (− 2.0, 5.7)	− 17.5 (− 22.9, − 11.7)^d^	− 5.4 (− 8.7, − 2.0)^d^
NT-proBNP								
Sleep	1.5 (− 1.6, 4.7)	2.2 (− 1.7, 6.3)	− 0.8 (− 3.9, 2.5)	− 2.0 (− 6.5, 2.6)	0.2 (− 3.5, 4.1)	0.6 (− 3.7, 5.0)	2.3 (− 0.3, 5.1)	1.8 (− 1.3, 5.0)
SB	− 1.8 (− 4.5, 1.0)	0.2 (− 2.9, 3.5)	5.8 (2.7, 8.9)^d^	− 0.6 (− 4.1, 3.0)	0.6 (− 3.2, 4.6)	− 0.7 (− 3.9, 2.6)	− 0.6 (− 3.0, 1.8)	− 1.7 (− 4.2, 0.8)
LPA	8.1 (0.5, 16.2)	− 1.2 (− 7.7, 5.7)	−19.3 (− 25.4, − 12.7)^d^	1.0 (− 5.8, 8.3)	− 1.6 (− 9.3, 6.7)	3.4 (− 2.6, 9.8)	− 4.9 (− 10.1, 0.7)	0.4 (− 4.3, 5.3)
MVPA	−6.7 (− 15.9, 3.4)	−4.8 (− 10.8, 1.6)	−23.1 (− 30.7, − 14.6)^d^	7.0 (− 0.9, 15.5)	−7.4 (− 19.8, 6.8)	− 2.1 (− 8.1, 4.3)	− 11.4 (− 20.9, − 0.8)	− 0.0 (− 5.8, 6.1)

Values are mean percentage differences^a^ (95% confidence interval) in each cardiac biomarker per 30 min/day increment in each movement behavior in each stratum

Linear regression models adjusted for sex, age, educational level (primary or less, secondary, or university), smoking status (never, former, or current), alcohol consumption (never, moderate, heavy, or former), energy intake (kcal/day), Mediterranean Diet Adherence Screener (MEDAS) score, body mass index (kg/m^2^), serum glucose (mg/dL), serum LDL-cholesterol (mg/dL), systolic blood pressure (mmHg) and glomerular filtration rate (mL/min)

^a^Mean percentage differences were calculated by subtracting 1 from the exponentiated β-coefficients in the regression models with log-transformed values of cardiac biomarkers and multiplying the result by 100

^b^Subclinical cardiac damage: hs-cTnT >p99 (16.8 pg/mL in men and 9.0 pg/mL in women) and/or NT-proBNP >cutoff (75 pg/mL if age ≤ 75 years, 250 pg/mL if age > 75 years)

^c^Low PA time: total PA time ≤ 3.53 h/day; high PA time: total PA time > 3.53 h/day

^d^Statistically significant association when using a false discovery rate of 5%. hs-cTnT: high-sensitivity cardiac troponin T; LPA: light physical activity; MVPA: moderate-to-vigorous physical activity; NT-proBNP: N-terminal pro-B-type natriuretic peptide; PA: physical activity; SB: sedentary behavior

Among women, no associations were found between movement behaviors and NT-proBNP levels, or between movement behaviors and hs-cTnT levels in those without subclinical cardiac damage. However, in women with subclinical cardiac damage, all movement behaviors except sleep were related with hs-cTnT levels, with differences according to the level of PA. Thus, spending more time in SB was linked to higher hs-cTnT levels only among the less active women with subclinical cardiac damage, with a MPD of 2.1 (0.7, 3.6) per 30 min/day increment (Table 2), and accumulating SB time in bouts ≥30 min did not modify much the association (Supplemental Table 3). Spending more time in LPA was related to lower hs-cTnT levels in the less active women with subclinical cardiac damage (− 5.1 [− 8.3, − 1.7] per 30 min/day increment in LPA), whereas in the more active ones, hs-cTnT levels were higher for more time in LPA (4.1 [1.2, 7.2] per 30 min/day increment) and for more sedentary breaks (9.4 [4.5, 14.6] per 1-SD increment) (Table 2, Supplemental Table 3). Spending more time in MVPA was associated with lower hs-cTnT levels among less active women with subclinical cardiac damage (MDP of − 17.5 [− 22.9, − 11.7] per 30 min/day), and also, although to a lesser extent, in the more active ones (− 5.4 [− 8.7, − 2.0]) (Table 2). When considering accumulated time in MVPA bouts ≥10 min, the association strengthened in the less active group (− 28.4 [− 41.1, − 13.0] per 30 min/day increment), but weakened in the more active one, becoming non-significant (Supplemental Table 3). Lastly, an inverse association was found between mean movement intensity and hs-cTnT levels only in less active women with subclinical cardiac damage (MDP per 1-SD increment of − 16.0 [− 22.2, − 9.4]) (Supplemental Table 3).

## Discussion

In our study of Spanish older adults without major CVD, the relationship between movement behaviors and cardiac biomarkers levels depends on sex, subclinical cardiac damage and PA level. The strongest associations were observed in less active individuals with subclinical cardiac damage, in whom more PA and less SB were generally related to lower levels of hs-cTnT and NT-proBNP.

Regular PA is one of the cornerstones of prevention and treatment of many chronic diseases, such as CHD [17], diabetes mellitus [40], hypertension [41] or obesity. However, in previous investigations, the dose-response relationship of more PA and less sedentariness with lower incidence of CVD [42] and lower all-cause mortality [15] in older adults was not linear. A meta-analysis on the effect of adherence to moderate-intensity PA (MPA) recommendations on the risk of CHD [18] showed that, compared with inactive participants, those performing the minimum recommended amount (150 min/week MPA) or the amount recommended for additional benefits (300 min/week MPA) had a 14% and a 20% lower risk of CHD, respectively. However, in those with higher PA, the risk reduction was only slightly higher than in those with 300 min/week MPA. Lastly, less active participants also had a significantly lower risk of CHD than the inactive ones, suggesting that doing some PA is better than doing nothing [43]. The non-linear associations of PA with cardiac biomarkers observed in our study in participants with subclinical cardiac damage are in line with this meta-analysis, as well as the lower levels of hs-cTnT found for more PA in less active women with subclinical cardiac damage and of both biomarkers in men. However, in contrast to previous studies that also reported a non-linear relationship between sleep and all-cause mortality and CVD, with the lowest risk for 6–8 h/day compared to short (< 6 h/day) [20] or long sleep duration (> 8–9 h/day) [20, 21], no evidence of a relationship with cardiac biomarker levels was found in our study.

Our results in men are also in line with those reported by Parsons et al. [27]. Like us, they observed a non-linear relationship of PA with cardiac biomarker levels in older men, and suggested that MVPA may be more important in protecting against cardiac health deterioration in less active men, consistent with the widely known health benefits of MVPA [17, 18]. However, while their results suggested that LPA could also play a role for hs-cTnT in less active men, ours support a more important role for NT-proBNP levels. SB also seemed to be more important in our study than in theirs for NT-proBNP levels at low levels of PA. Interestingly, in a post hoc analysis of the aforementioned study, movement behaviors were not associated with NT-proBNP among less active men with normal blood pressure, but only in those with hypertension, a group with higher NT-proBNP levels possibly consisting of individuals with subclinical cardiac damage, similar to our finding associations only among less active men with subclinical cardiac damage. The stronger associations of movement behaviors with NT-proBNP levels than with hs-cTnT levels in men with subclinical cardiac damage found in our study, and also reported by Parsons et al. [27], may be due to the different pathophysiological mechanisms involved in the production of each biomarker: cardiomyocyte injury for hs-cTnT and myocardial stretch for NT-proBNP [44].

The main difference between men and women in our study was the absence of associations for NT-proBNP observed in women. This may be explained by the stronger reported association of NT-proBNP with incident heart failure in men, and the stronger and earlier activation of the natriuretic peptide system in men [45, 46]. However, the associations with hs-cTnT observed in women and men in our study were consistent, although somewhat stronger in women, possibly due to the sex differences in PA intensity. Thus, although our findings among older women with subclinical cardiac damage who were more physically active, in whom more LPA and more sedentary breaks were linked to higher hs-cTnT, were unexpected, it is possible that when PA is already high, doing more LPA, which can mean also doing more sedentary breaks, does not add any benefit, possibly because it could even replace MVPA. In fact, using isotemporal substitution models, 30 min/day more of MVPA at the expense of LPA was associated with an 11.7% lower hs-cTnT level, suggesting that to obtain more benefits within the same PA time, the intensity of PA should be increased. However, given that men spend more time in MVPA and less in LPA than women (Supplementary Table 2), more active men with subclinical cardiac damage would not obtain any benefit from doing more PA, and less active men would obtain fewer benefits than less active women.

Regarding bouts, the most recent WHO PA guidelines [43] do not require PA to be performed in bouts of sufficient duration because new evidence shows that PA of any duration is associated with better health outcomes, including all-cause mortality [15] and multimorbidity [47]. In fact, we found that, among less active men with subclinical cardiac damage, the association with NT-proBNP did not vary much when MVPA time was accumulated in bouts ≥10 min, and the association with hs-cTnT was even lost. However, the fact that among less active women with subclinical cardiac damage (who perform very little MVPA, as shown in Supplemental Table 2) the association with hs-cTnT strengthened when MVPA time was accumulated in bouts ≥10 min suggests that the less active an individual is, the more important it is to increase the intensity of PA.

Cardiac biomarkers are also good indicators of CVD risk. A study in older men without CVD followed for 9 years has shown that a higher NT-proBNP was associated with an increased CVD risk [48]. Another investigation in a middle-aged European population over a 20-year follow-up reported that hs-cTnI is an independent predictor of CVD events, so those participants with hs-cTnI levels ≥12.7 pg/mL had 2.5 times the risk than those with non-detectable hs-cTnI levels. Interestingly, when cTnI was also measured by a high-sensitivity assay, the association remained significant even for those individuals with undetectable levels in the conventional assay [49]. Our results support that the association between movement behaviors and cardiac biomarkers depends on their baseline levels as well as the PA level and suggest that less active individuals with subclinical cardiac damage would obtain more benefits from moving more and sitting less. However, identifying the mechanisms involved in the benefits of movement behaviors on CVD through the improvement of cardiac biomarkers requires further research.

Our study has several strengths. In addition to the large sample size, the main strength is the use of accelerometry, which allowed objective assessment of different movement behaviors, including bouts, number of sedentary breaks or mean movement intensity. Another strength is that cTnT was determined by hs assays. Furthermore, although the analysis plan was not pre-registered, standard statistical procedures were used, specifically adjusting for potential confounders and stratifying by modifiers of the study associations, such as sex, level of PA and subclinical cardiac damage, to reduce the risk of bias. However, some limitations should be acknowledged. The main weakness is its cross-sectional design, which precludes making causal inferences. Wrist accelerometers have good wear-time compliance, but lower accuracy than those placed on the hip or the thigh, as they may not be able to distinguish between sitting and standing and may misclassify arm movements during sedentary activities as PA (particularly when used on the dominant wrist). Moreover, we did not check whether study participants wore the device on the non-dominant wrist all the time. Additionally, as the cardiac biomarkers were measured in frozen stored samples, the possibility of a variable decrease in concentration during long-term storage cannot be excluded, so some measurement error may have occurred. Also, as in any observational study, residual confounding may persist despite models were adjusted for many potential confounders. Furthermore, the large number of stratified analyses performed precluded additional stratification by age (e.g., below and above 75 years), because the resulting analyses would have insufficient statistical power to assess if age influenced the study results. Lastly, our results may not be generalizable to younger age groups, non-European populations, or even non-Mediterranean populations.

## Conclusions

In older men and women without major CVD, the link between movement behaviors and cardiac biomarkers depends on sex, subclinical cardiac damage and level of PA. Engaging in more activity and reducing sedentariness is generally more beneficial for participants who are initially less active and suffer subclinical cardiac damage, with greater benefits for hs-cTnT in women than in men and no benefit for NT-proBNP in women. These results support that changes in movement behaviors may contribute to lower CVD risk by reducing cardiac biomarkers levels, but they should be confirmed by prospective studies, or even randomized controlled trials aimed at investigating whether physical exercise interventions can reduce cardiac biomarker levels or prevent or delay further increases, given the scarcity of these studies [50, 51].

## Supplementary Information

**Additional file 1.** Supplemental tables 1–3 and Figs. 1-2.

## Data Availability

The datasets used and/or analyzed during the current study are available from the corresponding author on reasonable request.

## Abbreviations

hs-cTnT: high-sensitivity cardiac troponin T
NT-proBNP: N-terminal pro-B-type natriuretic peptide
PA: physical activity
SB: sedentary behavior
CVD: cardiovascular disease
LPA: light physical activity
MVPA: moderate-to-vigorous physical activity
p99: 99thpercentile
CHD: coronary heart disease
ENMO: Euclidean Norm of the raw accelerations Minus One
mg: milligravitational units
URL: Upper Reference Limit
MEDAS: Mediterranean Diet Adherence Screener
BMI: body mass index
SBP: systolic blood pressure
eGFR: estimated Glomerular Filtration Rate
CKD-EPI: Chronic Kidney Disease - Epidemiology Collaboration
MPD: mean percentage differences
