# Supplementary material for: Device-measured movement behaviors and cardiac biomarkers in older adults without major cardiovascular disease: the Seniors-ENRICA-2 study
Source: Eur Rev Aging Phys Act. 2023 Mar 9;20:5. doi: 10.1186/s11556-023-00313-8 (PMC9996928; doi:10.1186/s11556-023-00313-8)
Supplement: Supplementary file 1 — Additional file 1. Supplemental tables 1–3 and Figs. 1-2. [file 11556_2023_313_MOESM1_ESM.docx]

**Supplemental Table 1. Characteristics of study participants, by strata according to subclinical cardiac damage and PA time.**

|  | **Total** | **No subclinical cardiac damage^a^** | | **Subclinical cardiac damage^a^** | |
| --- | --- | --- | --- | --- | --- |
|  |  | **Low PA time^b^** | **High PA time^b^** | **Low PA time^b^** | **High PA time^b^** |
|  | n=1939 | n=438 | n=452 | n=532 | n=517 |
| **Sex, No. (%)** |  |  |  |  |  |
| Men | 860 (44.4) | 267 (61.0) | 234 (51.8) | 190 (35.7) | 169 (32.7) |
| Women | 1070 (55.7) | 171 (39.0) | 218 (48.2) | 342 (64.3) | 348 (67.3)* |
| **Age (years)** | 71.45 (4.24) | 71.77 (4.51) | 69.98 (3.74) | 72.88 (4.50) | 71.00 (3.59)* |
| **Educational level, No. (%)** |  |  |  |  |  |
| ≤ Primary | 1232 (63.5) | 248 (56.6) | 289 (63.9) | 343 (64.5) | 352 (68.1) |
| Secondary | 359 (18.5) | 97 (22.2) | 83 (18.4) | 90 (16.9) | 89 (17.2) |
| University | 348 (18.0) | 93 (21.2) | 80 (17.7) | 99 (18.6) | 76 (15.7)* |
| **Tobacco smoking, No. (%)** |  |  |  |  |  |
| Non-smoker | 1043 (53.8) | 202 (46.1) | 215 (47.6) | 316 (59.4) | 310 (60.0) |
| Former smoker | 713 (36.8) | 180 (41.1) | 200 (44.2) | 165 (31.0) | 169 (32.5) |
| Current smoker | 183 (9.4) | 56 (12.8) | 37 (8.2) | 51 (9.6) | 39 (7.5)* |
| **Alcohol consumption, No. (%)** |  |  |  |  |  |
| Non-drinker | 377 (19.4) | 76 (17.4) | 66 (14.6) | 132 (24.8) | 103 (19.9) |
| Former drinker | 115 (5.9) | 21 (4.8) | 22 (4.9) | 39 (7.3) | 33 (6.4) |
| Moderate drinker^c^ | 1010 (52.1) | 103 (23.5) | 124 (27.4) | 93 (17.5) | 117 (22.6) |
| Heavy drinker | 437 (22.5) | 238 (54.3) | 240 (53.1) | 268 (50.4) | 264 (51.1)* |
| **MEDAS score (0-14)** | 7.16 (1.72) | 7.02 (1.73) | 7.39 (1.78) | 7.06 (1.67) | 7.18 (1.69)* |
| **Energy intake (kcal/day)** | 1936 (345) | 1965 (330) | 2015 (375) | 1880 (347) | 1899 (309)* |
| **BMI (kg/m^2^), No. (%)** |  |  |  |  |  |
| <25 | 540 (27.8) | 103 (23.5) | 139 (30.5) | 127 (23.9) | 172 (33.3) |
| 25-30 | 934 (48.2) | 227 (51.8) | 237 (52.4) | 232 (43.6) | 238 (46.0) |
| ≥30 | 465 (24.0) | 108 (24.7) | 77 (17.1) | 173 (32.5) | 107 (20.7)* |
| **SBP (mmHg)** | 134.6 (18.2) | 134.1 (16.1) | 132.5 (16.1) | 136.9 (19.5) | 134.1 (19.0)* |
| **Serum glucose (mg/dL)** | 98.6 (22.7) | 101.8 (24.0) | 98.2 (21.6) | 100.4 (27.0) | 94.4 (16.2)* |
| **Serum LDL-cholesterol (mmol/L)** | 1.32 (0.47) | 1.27 (0.45) | 1.37 (0.48) | 1.28 (0.45) | 1.34 (0.48)* |

Values are means (standard deviations) unless indicated. * p<0.05 for differences across strata. BMI: body mass index; eGFR: Estimated Glomerular Filtration Rate; MEDAS Mediterranean Diet Adherence Screener; PA: physical activity; SBP: systolic blood pressure.

^a^Subclinical cardiac damage: hs-cTnT >p99 (16.8pg/mL in men and 9.0pg/mL in women) and/or NT-proBNP >cutoff (75 pg/mL if aged 50-75 years, 250 pg/mL if age >75 years).

^b^Low PA time: total PA time ≤3.53 h/day; high PA time: total PA time >3.53 h/day.

^c^Moderate drinker: <10 g/day in women and <20 g/day in men.

^d^Estimated Glomerular Filtration Rate by the CKD-EPI (Chronic Kidney Disease Epidemiology Collaboration) equation.

**Supplemental Table 2. Time spent in each movement behavior in men and women, stratified by subclinical cardiac damage and total PA time.** Values are means (standard deviations).

|  |  | **Men** | | | | | | | |
| --- | --- | --- | --- | --- | --- | --- | --- | --- | --- |
|  |  | **No subclinical cardiac damage^a^** | | |  | **Subclinical cardiac damage^a^** | | |  |
|  |  | **Low PA time^b^** |  | **High PA time^b^** |  | Low PA time^b^ |  | **High PA time^b^** |  |
|  |  | n=267 |  | n=234 |  | n=190 |  | n=169 |  |
| **Sleep** (hours/day) |  | 7.74 (1.09) |  | 7.42 (0.92) |  | 7.80 (1.25) |  | 7.32 (0.91) |  |
| **SB** (hours/day) |  | 13.48 (1.20) |  | 11.97 (1.13) |  | 13.55 (1.35) |  | 11.92 (1.18) |  |
| **LPA** (min/day) |  | 111.83 (27.53) |  | 171.06 (31.22) |  | 107.09 (29.95) |  | 179.39 (35.94)**^†^** |  |
| **MVPA** (min/day) |  | 44.66 (19.53) |  | 95.75 (32.67) |  | 42.71 (22.93) |  | 96.13 (32.74) |  |
|  |  | **Women** | | | | | | | |
|  |  | **No subclinical cardiac damage^a^** | | |  | **Subclinical cardiac damage^a^** | | |  |
|  |  | **Low PA time^b^** |  | **High PA time^b^** |  | Low PA time^b^ |  | **High PA time^b^** |  |
|  |  | n=171 |  | n=218 |  | n=342 |  | n=348 |  |
| **Sleep** (hours/day) |  | 7.93 (1.10) |  | 7.44 (0.85) |  | 8.01 (1.13)* |  | 7.57 (0.95)* |  |
| **SB** (hours/day) |  | 13.24 (1.08)* |  | 11.65 (1.14)* |  | 13.27 (1.23)* |  | 11.68 (1.14)* |  |
| **LPA** (min/day) |  | 125.44 (30.84)* |  | 198.19 (36.81)* |  | 122.63 (31.20)* |  | 199.14 (36.72)* |  |
| **MVPA** (min/day) |  | 34.40 (17.64)* |  | 85.77 (35.05)* |  | 31.31 (15.95)* |  | 77.19 (29.82)***^†^** |  |

*p<0.05 for differences between men and women in the same stratum. ^†^p<0.05 for differences between participants with and without subclinical cardiac damage in the same category of PA time. LPA = light physical activity; MVPA = moderate-to-vigorous physical activity; PA = physical activity; SB = sedentary behavior.

^a^ Subclinical cardiac damage: cTnT-hs > p99 (16.8 pg/mL in men and 9.0 pg/mL in women) and/or NT-proBNP > cutoff (75 pg/mL if age ≤ 75 years, 250 pg/mL if age > 75 years).

^b^ Low PA time: total PA time ≤3.53 h/day; high PA time: total PA time >3.53 h/day.

**Supplemental Table 3. Association of other accelerometry variables with cardiac biomarkers in men and women, stratified by subclinical cardiac damage and PA time**. Values are mean percentage differences^a^ (95% CI) in each cardiac biomarker per 30 min/day increment in time spent in bouts of movement behaviors or per 1-SD increment in the number of sedentary breaks and mean movement intensity in each stratum.

|  | Men | | | |  | Women | | | |
| --- | --- | --- | --- | --- | --- | --- | --- | --- | --- |
|  | **No subclinical**  **cardiac damage^b^** | | **Subclinical**  **cardiac damage^b^** | |  | **No subclinical**  **cardiac damage^b^** | | **Subclinical**  **cardiac damage^b^** | |
|  | **Low PA time^c^**  n=267 | **High PA time^c^**  n=234 | **Low PA time^c^**  n=190 | **High PA time^c^**  n=169 |  | **Low PA time^c^** n=171 | **High PA time^c^** n=218 | **Low PA time^c^**  n=342 | **High PA time^c^**  n=348 |
| hs-cTnT |  |  |  |  |  |  |  |  |  |
| Time in sedentary bouts ≥30 min | -0.2 (-1.3, 0.8) | -0.5 (-1.8, 0.8) | 2.7 (1.5, 3.9)* | -1.1 (-2.4, 0.3) |  | -0.7 (-2.1, 0.6) | -0.6 (-1.9, 0.7) | 1.6 (0.6, 2.5)* | -0.1 (-1.1, 0.9) |
| Number of sedentary breaks | 4.3 (-1.1, 10.0) | 3.1 (-3.1, 9.7) | -2.7 (-8.9, 3.9) | 5.3 (-1.7, 12.7) |  | 1.7 (-4.8, 8.8) | 1.3 (-4.6, 7.6) | -4.8 (-9.3, -0.1) | 9.4 (4.5, 14.6)* |
| Time in MVPA bouts ≥10 min | -3.4 (-14.1, 8.7) | 1.3 (-4.0, 6.9) | -6.8 (-17.2, 5.0) | 2.0 (-4.5, 8.8) |  | 14.3 (-4.9, 37.4) | 1.2 (-5.5, 8.4) | -28.4 (-41.1, -13.0)* | -4.6 (-11.7, 3.0) |
| Mean movement intensity (mg) | -0.3 (-6.2, 6.0) | 1.1 (-3.8, 6.1) | -7.0 (-13.5, -0.0) | 4.2 (-1.7, 10.4) |  | 7.2 (-2.6, 18.0) | 4.0 (-2.0, 10.4) | -16.0 (-22.2, -9.4)* | -1.6 (-6.4, 3.5) |
| NT-proBNP |  |  |  |  |  |  |  |  |  |
| Time in sedentary bouts ≥30 min | -1.4 (-3.1, 0.3) | 0.4 (-1.7, 2.6) | 5.6 (3.6, 7.7)* | -1.0 (-3.2, 1.3) |  | 0.2 (-2.1, 2.5) | 0.7 (-1.5, 2.8) | 0.7 (-0.8, 2.3) | -1.2 (-2.9, 0.6) |
| Number of sedentary breaks | 7.1 (-1.9, 17.1) | -2.4 (-11.9, 8.1) | -25.4 (-33.1, -16.8)* | 2.5 (-8.4, 14.7) |  | -3.5 (-13.6, 7.8) | -1.0 (-10.3, 9.4) | -5.8 (-13.0, 2.0) | 2.8 (-4.8, 10.9) |
| Time in MVPA bouts ≥10 min | -16.2 (-31.0, 1.9) | -7.2 (-15.1, 1.5) | -23.6 (-37.2, -7.0)* | 4.4 (-6.3, 16.3) |  | -1.7 (-27.6, 33.5) | 1.2 (-9.6, 13.5) | -8.0 (-33.4, 27.2) | 4.0 (-8.5, 18.1) |
| Mean movement intensity (mg) | -7.4 (-16.3, 2.4) | -4.5 (-11.9, 3.6) | -27.8 (-35.9, -18.6)* | 4.1 (-5.5, 14.6) |  | -7.3 (-21.0, 8.7) | -0.6 (-10.0, 9.7) | -14.2 (-24.3, -2.6) | 2.1 (-6.1, 11.0) |

*Statistically significant association when using a false discovery rate of 5%. hs-cTnT: high-sensitivity cardiac troponin T; MVPA: moderate-to-vigorous physical activity; NT-proBNP: N-terminal pro-B-type natriuretic peptide; PA: physical activity.

^a^Mean percentage differences were calculated by subtracting 1 from the exponentiated β-coefficients in the regression models with log-transformed values of cardiac biomarkers and multiplying the result by 100.

^b^Subclinical cardiac damage: hs-cTnT >p99 (16.8 pg/mL in men and 9.0 pg/mL in women) and/or NT-proBNP >cutoff (75 pg/mL if age ≤75 years, 250 pg/mL if age >75 years).

^c^Low PA time: total PA time ≤3.53 h/day; high PA time: total PA time >3.53 h/day.

Linear regression models adjusted for sex, age, educational level (primary or less, secondary, or university), smoking status (never, former, or current), alcohol consumption (never, moderate, heavy, or former), energy intake (kcal/day), Mediterranean Diet Adherence Screener (MEDAS) score, body mass index (kg/m^2^), serum glucose (mg/dL), serum LDL-cholesterol (mg/dL), systolic blood pressure (mmHg) and glomerular filtration rate (mL/min).

**Supplemental Figure 1.** **Association of each movement behavior with hs-cTnT in men and women without subclinical cardiac damage**. Restricted cubic splines whose values are geometric means (95%-confidence interval) of hs-cTnT.

hs-cTnT: high-sensitivity cardiac troponin T; LPA: light physical activity; MVPA: moderate-to-vigorous physical activity; PA: physical activity; SB: sedentary behavior.

Subclinical cardiac damage: hs-cTnT >p99 (16.8pg/mL in men and 9.0pg/mL in women) and/or NT-proBNP >cutoff (75pg/mL if age ≤75 years, 250pg/mL if age >75 years).

Linear regression models adjusted for sex, age, educational level (primary or less, secondary, or university), smoking status (never, former, or current), alcohol consumption (never, moderate, heavy, or former), energy intake (kcal/day), Mediterranean Diet Adherence Screener (MEDAS) score, body mass index (kg/m^2^), serum glucose (mg/dL), serum LDL-cholesterol (mg/dL), systolic blood pressure (mmHg) and glomerular filtration rate. Models for sleep and SB further adjusted for MVPA time, and models for LPA and MVPA further adjusted for SB time.

**Supplemental table 2.** **Association of each movement behavior with NT-proBNP in men and women without subclinical cardiac damage**.

Restricted cubic splines whose values are geometric means (95%-confidence interval) of NT-proBNP.

LPA: light physical activity; MVPA: moderate-to-vigorous physical activity; NT-proBNP: N-terminal pro-B-type natriuretic peptide; PA: physical activity; SB: sedentary behavior.

Subclinical cardiac damage: hs-cTnT >p99 (16.8pg/mL in men and 9.0pg/mL in women) and/or NT-proBNP >cutoff (75pg/mL if age ≤75 years, 250pg/mL if age >75 years).

Linear regression models adjusted for sex, age, educational level (primary or less, secondary, or university), smoking status (never, former, or current), alcohol consumption (never, moderate, heavy, or former), energy intake (kcal/day), Mediterranean Diet Adherence Screener (MEDAS) score, body mass index (kg/m^2^), serum glucose (mg/dL), serum LDL-cholesterol (mg/dL), systolic blood pressure (mmHg) and glomerular filtration rate. Models for sleep and SB further adjusted for MVPA time, and models for LPA and MVPA further adjusted for SB time.
